# A Novel Function for 15-Lipoxygenases in Cholesterol Homeostasis and CCL17 Production in Human Macrophages

**DOI:** 10.3389/fimmu.2018.01906

**Published:** 2018-08-24

**Authors:** Ryan G. Snodgrass, Ekaterina Zezina, Dmitry Namgaladze, Sahil Gupta, Carlo Angioni, Gerd Geisslinger, Dieter Lütjohann, Bernhard Brüne

**Affiliations:** ^1^Faculty of Medicine, Institute of Biochemistry I, Goethe-University Frankfurt, Frankfurt, Germany; ^2^ZAFES/Pharmazentrum Frankfurt, Institute of Clinical Pharmacology, Goethe-University Frankfurt, Frankfurt, Germany; ^3^Fraunhofer Institute for Molecular Biology and Applied Ecology IME, Branch for Translational Medicine and Pharmacology TMP, Frankfurt, Germany; ^4^Institute for Clinical Chemistry and Clinical Pharmacology, University of Bonn, Bonn, Germany

**Keywords:** macrophage, arachidonate 15-lipoxygenase, type B, lipoxygenase, chemokine, cholesterol, interleukin-4, sterol regulatory element binding protein-2

## Abstract

Arachidonate 15-lipoxygenase (ALOX15) and arachidonate 15-lipoxygenase, type B (ALOX15B) catalyze the dioxygenation of polyunsaturated fatty acids and are upregulated in human alternatively activated macrophages (AAMs) induced by Th2 cytokine interleukin-4 (IL-4) and/or interleukin-13. Known primarily for roles in bioactive lipid mediator synthesis, 15-lipoxygenases (15-LOXs) have been implicated in various macrophage functions including efferocytosis and ferroptosis. Using a combination of inhibitors and siRNAs to suppress 15-LOX isoforms, we studied the role of 15-LOXs in cellular cholesterol homeostasis and immune function in naïve and AAMs. Silencing or inhibiting the 15-LOX isoforms impaired sterol regulatory element binding protein (SREBP)-2 signaling by inhibiting SREBP-2 processing into mature transcription factor and reduced SREBP-2 binding to sterol regulatory elements and subsequent target gene expression. Silencing ALOX15B reduced cellular cholesterol and the cholesterol intermediates desmosterol, lanosterol, 24,25-dihydrolanosterol, and lathosterol as well as oxysterols in IL-4-stimulated macrophages. In addition, attenuating both 15-LOX isoforms did not generally affect IL-4 gene expression but rather uniquely impacted IL-4-induced CCL17 production in an SREBP-2-dependent manner resulting in reduced T cell migration to macrophage conditioned media. In conclusion, we identified a novel role for ALOX15B, and to a lesser extent ALOX15, in cholesterol homeostasis and CCL17 production in human macrophages.

## Introduction

Macrophages are versatile immune cells with a broad functional repertoire of sensors allowing them to respond to a variety of environmental cues and acquire diverse but specialized functional phenotypes. Stimulation with Th2 cytokines interleukin-4 (IL-4) or interleukin-13 induces a distinct gene expression pattern and phenotype termed alternative activation (1–3). In human alternatively activated macrophages (AAMs), both 15-lipoxygenase (LOX) isoforms arachidonate 15-lipoxygenase (ALOX15) and arachidonate 15-lipoxygenase, type B (ALOX15B) are upregulated (1–3). The ALOX15 and ALOX15B encoded non-heme-iron-containing enzymes catalyze the dioxygenation of polyunsaturated fatty acids (PUFAs), which as the names suggests, insert molecular oxygen at carbon atom 15 of arachidonic acid to generate 15-hydroperoxyeicosatetraenoic acid [15(S)-HpETE] which is rapidly reduced by glutathione peroxidases to 15-hydroxyeicosatetraenoic acid [15(S)-HETE] (4). Many of the hydroperoxy fatty acids produced by 15-LOXs can be further metabolized by various enzyme families to produce an array of specialized pro-resolving mediators including lipoxins, protectins, and resolvins (5). In addition to metabolizing free PUFAs hydrolyzed from phospholipids, 15-LOXs directly oxidize PUFAs esterified to membrane-bound phospholipids as well as within cholesterol esters (CEs) (6–8).

Although the biological significance of endogenously generated esterified eicosanoids in phospholipids is not well understood, the ability of 15-LOXs to modify membranes of non-immune cells has been previously described in reticulocyte maturation where ALOX15 facilitates mitochondria breakdown during late erythropoiesis by oxidizing mitochondrial membrane lipids (9). In macrophages, up to 1.5% of the total cellular phosphatidylethanolamine (PE) pool is reported to contain 15(S)-HETE (7). Macrophages derived from 12/15-lipoxygenase-deficient mice showed altered cell membrane structure, increased levels of PE and phosphatidylinositol, reduced levels of phosphatidycholine (PC), and displayed defects in autophagy (10). Moreover, 12/15-lipoxygenase-deficient macrophages are unable to fully phagocytose apoptotic cells, further supporting a role for 15-LOXs in processes involving membrane remodeling (11).

Previous publications have also reported cholesterol-modifying and mobilizing activities of 15-LOX in macrophages. ALOX15 has been shown to oxygenate intracellular CEs, which are preferentially hydrolyzed over their nonoxidized counterparts by CE hydrolases (12) leading to reacylation of the released oxidized fatty acyl chains into PCs forming oxidized PCs (13). When overexpressed in immortalized macrophages, ALOX15 increased CE hydrolase activity and reduced intracellular CE accumulation in cholesterol-loaded cells (8, 14). With respect to cholesterol, a majority of a cell’s total cholesterol is distributed within the plasma membrane (PM) where it associates with various phospholipids and sphingolipids forming dynamic complexes with specific stoichiometries. Altering membrane phospholipid levels directly affects the distribution of cholesterol in cellular membranes (15, 16) which can vary by as much as 10-fold depending on the abundance of various phospholipids (16). In addition, reducing the sphingomyelin content of PM redistributes cholesterol from PM to ER (17). These findings raise the question of whether membrane remodeling processes attributed to 15-LOXs also influence cellular cholesterol homeostasis.

In the present study, we used human AAMs, which express both 15-LOX isoforms, to investigate their roles in macrophage cholesterol homeostasis and immune function. Using a combination of inhibitors and siRNAs to suppress the 15-LOX isoforms, we show that ALOX15B, and to a lesser extent ALOX15, regulate cholesterol levels in human macrophages in a sterol regulatory element binding protein (SREBP)-2-dependent manner. We also show that attenuating 15-LOXs does not generally affect IL-4 gene expression but rather uniquely impacts IL-4-induced CCL17 production thereby supporting a role for SREBP-2 in the immune response of AAMs.

## Experimental Procedures

### Reagents

ML351, PD146176, 25-hydroxycholesterol, 15(S)-HETE, 13(S)-HODE, and 17(S)-HDHA were purchased from Cayman Chemical (Ann Arbor, MI, USA), Bafilomycin A1, Methyl-β-cyclodextrin from Sigma-Aldrich (Steinheim, Germany), CC chemokine receptor 4 (CCR4) antagonist from EMD Millipore Corp. (Billerica, MA, USA), and recombinant human IL-4 from PeproTech (Hamburg, Germany). Primers were purchased from http://Biomers.net GmbH (Ulm, Germany) and their sequences are available on request. All chemicals were of the highest grade of purity and commercially available.

### Primary Human Macrophage Generation

Human monocytes were isolated from commercially obtained buffy coats from anonymous donors (DRK-Blutspendedienst Baden-Württemberg-Hessen, Institut für Transfusionsmedizin und Immunhämatologie, Frankfurt, Germany) using Ficoll density centrifugation. Peripheral blood mononuclear cells were washed twice with PBS containing 2 mM EDTA and subsequently incubated for 1 h at 37°C in RPMI 1640 medium supplemented with penicillin (100 U/ml) and streptomycin (100 µg/ml) to allow their adherence to culture dishes (Sarstedt, Nümbrecht, Germany). Nonadherent cells were removed. Monocytes were then differentiated into naïve macrophages with RPMI 1640 medium containing 5% AB-positive human serum (DRK-Blutspendedienst Baden-Württemberg-Hessen, Frankfurt, Germany) for 7 days.

### siRNA Transfections

Control siRNA and siRNAs targeting human ALOX15, ALOX15B, and SREBP2 (siRNA; siGENOME human SMARTpool, Thermo Scientific, Karlsruhe, Germany) were transfected into primary human macrophages at a final concentration of 50 nM using HiPerFect transfection reagent (Qiagen, Hilden, Germany) according to the manufacturer’s recommendations. Each knockdown (KD) was routinely confirmed by qRT-PCR for each experiment.

### RNA Extraction and Quantitative Real-Time PCR

Total RNA from macrophages was isolated using PeqGOLD RNAPure reagent (PeqLab Biotechnology, Erlangen, Germany) and quantified using the NanoDrop spectrophotometer (NanoDrop, Wilmington, DE, USA). Total RNA (1 µg) was transcribed with the Maxima First Strand cDNA Synthesis Kit (Fermentas, St. Leon-Rot, Germany). Quantitative real-time PCR was performed using iQ SYBR green Supermix (Bio-Rad Laboratories, Munich, Germany) and the Bio-Rad CFX96 system. Primer sequences for quantitative PCR can be obtained upon request. β2 microglobulin (BMG) was used as an endogenous control for human macrophages.

### Immunoblots

Macrophages lysates were resolved on polyacrylamide gels followed by transfer onto nitrocellulose membranes. Membranes were blocked with 5% milk/100 mM Tris–HCl, 150 mM sodium chloride, 0.01% (v/v) Tween 20 (TTBS) followed by incubation with antibodies against ALOX15 (Abcam, Cambridge, UK), ALOX15B (Cayman Chemical), ALOX5 (BD Biosciences), SREBP-2 (Cayman Chemical), or Nucleolin (Santa Cruz Biotechnology). For protein detection, the membrane was incubated with IRDye secondary antibodies (LI-COR Biosciences, Bad Homburg, Germany) in 5% BSA/TTBS. Proteins were visualized and when applicable densitometrically analyzed with the Odyssey infrared imaging system (LI-COR Biosciences).

### Enzyme-Linked Immunosorbent Assay

Macrophage supernatants were collected, briefly centrifuged to pellet cell debris, then analyzed for human CCL17 using LEGEND MAX ELISA kit (BioLegend, San Diego, CA, USA) according to the manufacturer’s recommended procedures.

### Cell Migration Assays

For cell migration assays, 2 × 10^6^ HUT78 cells were suspended in serum-free RPMI medium in Transwell permeable inserts (24-well, 5.0-µm polycarbonate membrane, upper compartment; Costar; Corning, Kennebunk, ME, USA) and allowed to migrate toward serum-free RPMI medium or macrophage conditioned media (CM) in the lower compartment for 14 h at 37°C. Macrophage CM was prepared as follows. Macrophages were left untreated or stimulated with IL-4 alone or in combination with ML351 or PD146176 in RPMI 1640 medium containing 5% AB-positive human serum for 48 h. Macrophage media was then collected, briefly centrifuged, and diluted with serum-free RPMI (1:1). In some experiments, HUT78 cells were treated with CCR4 antagonist (1.0 or 0.5 µM) for 20 min prior to use in migration assay. Following chemotaxis, migrated HUT78 cells were collected from lower chamber and quantified by flow cytometry. Chemotactic index was calculated as the fold increase in migration observed over the migration to CM from untreated macrophages (untreated Mφ CM).

### Chromatin Immunoprecipitation (ChIP)

Macrophages were fixed in 1% paraformaldehyde, quenched with 0.125 M glycine, and washed in PBS. Cells were lysed (10 mM HEPES/KOH pH 7.9, 1 mM EDTA, 85 mM KCl, 0.5% NP-40) to release cytosolic proteins and debris and the nuclear pellet was then lysed in nuclei lysis buffer (50 mM Tris–HCl pH 7.4, 10 mM EDTA, 1% SDS, 0.5% Empigen BB) and sonified with Branson Sonifier. Soluble chromatin was diluted with dilution buffer (0.5% Trition X-100, 2 mM EDTA, 20 mM Tris–HCl pH 7.4, 100 mM NaCl). Lysate were pre-cleared with sepharose CL-4B beads (Sigma-Aldrich) for 1 h and 1% of input was stored at 4°C. Chromatin was incubated with SREBP-2 (kindly provided by Dr. Timothy F. Osborne, Sanford-Burnham Medical Research Institute) or IgG (Abcam) antibodies overnight at 4°C then captured with Protein A/G beads (Santa Cruz Biotechnology). Antibody/bead complexes were washed once with low salt buffer (0.1% SDS, 1% Triton X-100, 2 mM EDTA, 20 mM Tris–HCl pH 7.4, 150 mM NaCl), once with high salt buffer (0.1% SDS, 1% Triton X-100, 2 mM EDTA, 20 mM Tris–HCl pH 7.4, 500 mM NaCl) and twice with LiCl buffer (250 mM LiCl, 10 mM Tris–HCl, pH 7.4, 1% NP-40, 1% sodium deoxycholate, 1 mM EDTA). The beads were eluted in 200 µl of EB (100 mM NaHCO_3_, 1% SDS) and the eluate was reverse crosslinked with RNAse and treated with proteinase K at 65°C for 4 h. The decrosslinked DNA was then purified using Qiagen Ampure purification kit (Qiagen) and eluted in 100 µl of elution buffer.

### Flow Cytometry

Macrophages were harvested, washed with PBS, then pelleted at 300 × *g* at 4°C for 5 min. Cells were blocked with 2% Fc Receptor Binding Inhibitor (eBioscience, Frankfurt, Germany) in PBS for 10 min on ice. Afterward, the following antibody mix was added in 100 ml PBS: anti-CD80-APC, anti-CD86-FITC (BD Biosciences, Heidelberg, Germany), anti-CD163-PE, and anti-CD206-PE-Cy5 (BioLegend, San Diego, CA, USA), followed by incubation for 20 min on ice in the dark. Samples were washed and analyzed by flow cytometry using an LSRII Fortessa cell analyzer (BD Biosciences).

### Measurement of Cellular Cholesterol, Non-Cholesterol Sterol, and Oxysterol Content

In a first series of experiments total, free, and esterified macrophage cholesterol was analyzed using the Amplex Red Cholesterol Assay Kit (Life Technologies) according to the manufacturer’s recommended procedures. Total cholesterol was measured in the presence of cholesterol esterase while free cholesterol was measured in the absence of cholesterol esterase in the reaction. CEs were determined by subtracting the value of free cholesterol from total cholesterol. Fluorescence was measured in a TECAN Infinite 200 PRO microplate reader (TECAN, Männedorf, Switzerland) using excitation of 545 nm and emission of 590 nm. Macrophage protein concentrations were measured using the DC Protein Assay (Bio-Rad Laboratories) and cholesterol concentrations was reported per μg protein.

In a second series of experiments, cholesterol, non-cholesterol sterol, and oxysterol content was analyzed by gas chromatography (GC). Macrophage cell pellets were spun in a speedvac concentrator (12 mbar; Savant AES 1000) and weighed. Cholesterol, non-cholesterol sterols, and oxysterols were extracted using chloroform. After alkaline hydrolysis the concentrations of cholesterol precursors were measured with GC-mass spectrometry-selected ion monitoring (18). The trimethylsilyethers of the sterols were separated on a DB-XLB (30 m length × 0.25 mm internal diameter, 0.25 µm film) column (Agilent Technologies, Waldbronn, Germany) using the 6890N Network GC system (Agilent Technologies). Epicoprostanol (Steraloids, Newport, RI, USA) was used as an internal standard, to quantify the non-cholesterol sterols (Medical Isotopes, Pelham, NH, USA) on a 5973 Network MSD (Agilent Technologies). Total cholesterol was measured by GC-flame ionization detection on an HP 6890 GC system (Hewlett Packard, Waldbronn, Germany), equipped with a DB-XLB (30 m length × 0.25 mm internal diameter, 0.25 µm film) column (Agilent Technologies) using 5a-cholestane (Steraloids) as internal standard (19).

### Lipid Analysis

5(S)-HETE, 12(S)-HETE, 13(S)-HODE, and 15(S)-HETE in the extracted samples were analyzed employing LC-MS/MS. The LC/MS-MS system comprised a 5500 QTrap mass spectrometer (Sciex, Darmstadt, Germany), an Agilent 1200 binary HPLC pump (Agilent Technologies), and an HTC Pal autosampler (Chromtech, Bad Camberg, Germany). Eicosanoid standards were obtained from Cayman Chemical. Sample extraction was performed with liquid–liquid extraction using ethyl acetate. The organic phase was removed under a stream of nitrogen, and the residues were reconstituted in 50 µl methanol/water/BHT (50:50:10^−4^, v/v/v) prior to injection into the LC-MS/MS system. For chromatographic separation, a Gemini NX C18 column and precolumn were used (Phenomenex, Aschaffenburg, Germany). A linear gradient was employed at a flow rate of 0.5 ml/min with a total run time of 17.5 min. The mobile phases were (A) water/ammonia (100:0.01, v/v) and (B) acetonitrile/ammonia (100:0.01, v/v). Retention times of 5(S)-HETE, 12(S)-HETE, 15(S)-HETE, and 13-HODE were 8.31, 7.41, 6.97, and 6.40 min, respectively. Peak quantification was performed with Multiquant software version 3.0.2 (Sciex) employing the internal standard method (isotope dilution mass spectrometry). The ratios of analyte peak area and internal standard area (*y*-axis) were plotted against concentration (*x*-axis), and calibration curves were calculated by least square regression with 1/square concentration weighting.

### Statistical Analysis

Graphical data are presented as mean ± SE of at least three independent experiments. *P*-values were calculated using one-way ANOVA with Bonferroni *post hoc* means comparisons or two-tailed Student’s *t*-test with significance level set at 0.05 (GraphPad, La Jolla, CA, USA). Pairwise correlations of gene expression in bronchial alveolar lavage (BAL) cells were performed using the R project software package.^1^

### Ethics

Investigations were conducted in accordance with the ethical standards and according to the Declaration of Helsinki and to the national and international guidelines and have been approved by the authors’ institutional review board.

## Results

### Lipoxygenase Expression and Activity in IL-4-Stimulated Primary Human Macrophages

**Figure 1:**
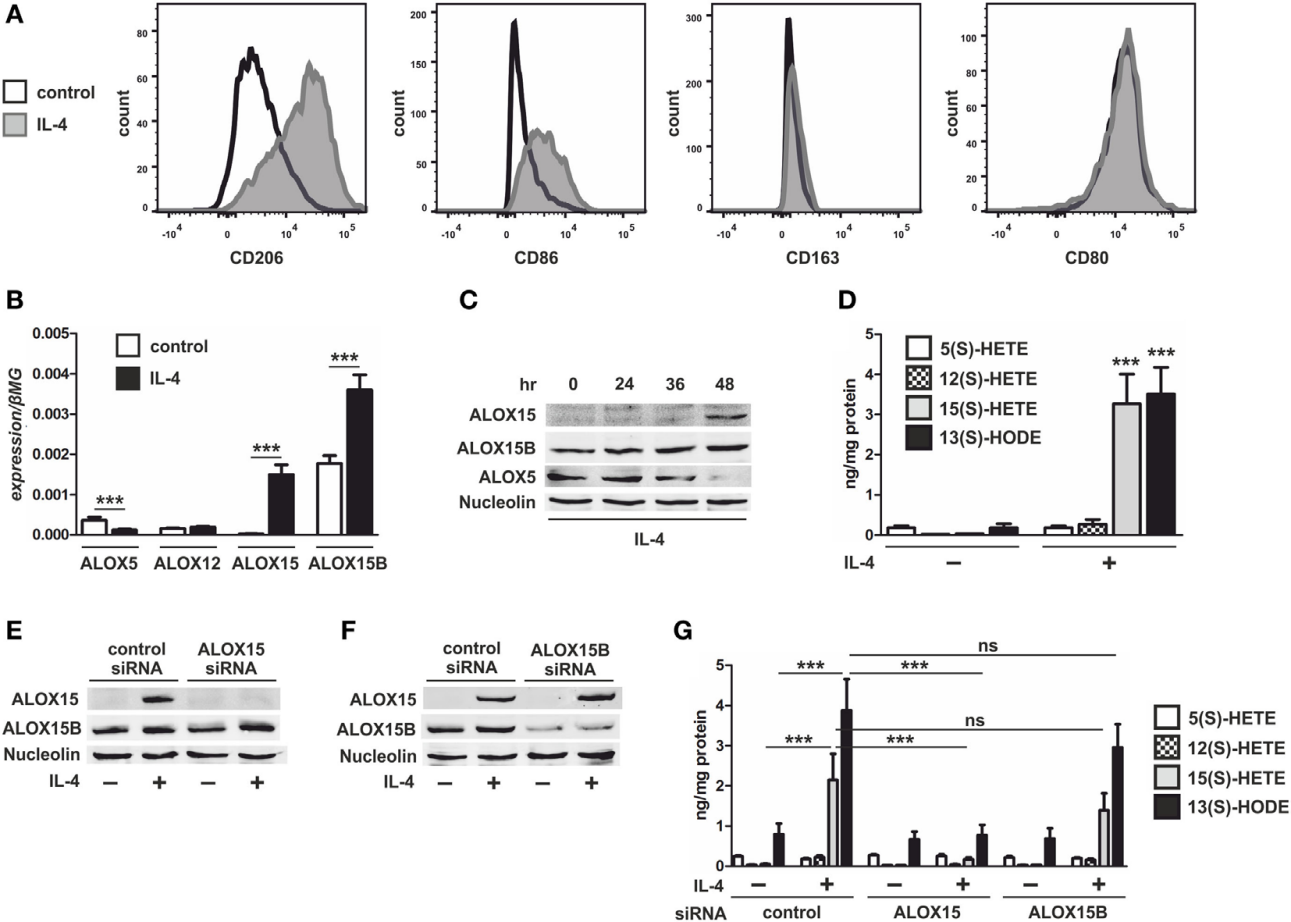
Lipoxygenase expression in interleukin-4 (IL-4)-stimulated macrophages. **(A)** Flow cytometric analysis of CD206, CD86, CD163, and CD80 **(B)** and mRNA expression of 5-lipoxygenase (ALOX5), 12-lipoxygenase (ALOX12), 15-lipoxygenase (ALOX15), and 15-lipoxygenase, type B (ALOX15B) in primary human macrophages treated with 20 ng/ml IL-4 for 48 h. *P*-values were calculated using two-tailed Student’s *t*-test. **(C)** Western analysis of ALOX15, ALOX15B, and ALOX5 expression in macrophages treated for indicated times with IL-4. **(D)** LC-MS/MS analysis of arachidonic acid metabolites 5-HETE, 12-HETE, 15-HETE, and linoleic acid (LA) metabolite 13-HODE in macrophages treated for 48 h with IL-4. *P*-values were calculated using two-tailed Student’s *t*-test. **(E,F)** Western analysis of ALOX15 and ALOX15B expression in macrophages transfected with control and ALOX15 **(D)** or ALOX15B **(E)** siRNAs 24 h prior to treatment with IL-4 for 48 h. **(G)** LC-MS/MS analysis of arachidonic acid metabolites 5-HETE, 12-HETE, 15-HETE, and LA metabolite 13-HODE in macrophages transfected with control, ALOX15, or ALOX15B siRNAs 24 h prior to treatment with IL-4 for 48 h. *P*-values were calculated using one-way ANOVA with Bonferroni *post hoc* means comparisons with significance level set at 0.05. ***P < 0.001. Data are mean values ± SE of at least three independent experiments.

To evaluate the role of 15-LOXs in macrophage cholesterol homeostasis we first assessed the expression of 15-LOX isoforms ALOX15 and ALOX15B in naïve and IL-4-induced AAMs known to express both 15-LOX isoforms (1–3, 20). IL-4 stimulation strongly increased the AAM cell surface marker CD206, slightly increased CD86 but not CD163 or CD80 (Figure 1A), and increased mRNA levels of ALOX15 and ALOX15B in human monocyte-derived macrophages (Figure 1B). The expression of additional lipoxygenase-isoforms expressed in human macrophages, including ALOX12 remained at the same low level as in unstimulated macrophages, while ALOX5 was reduced following IL-4 stimulation (Figure 1B). While both ALOX15 and ALOX15B expression increased following IL-4 stimulation, only ALOX15B mRNA was expressed in naïve macrophages. Western analysis confirmed expression of ALOX15B protein in unstimulated macrophages and the induction of ALOX15 and ALOX15B in a time-dependent manner following addition of IL-4 (Figure 1C). ALOX5 protein expression was suppressed following IL-4 stimulation. To assess 15-LOX activity we used LC-MS/MS to measure arachidonic acid and linoleic acid metabolites, hydroxyeicosatetraenoic acids (HETEs), and hydroxyoctadecadienoic acids (HODEs), respectively. Following IL-4 stimulation cellular levels of 15(S)-HETE and 13(S)-HODE strongly increased, while 12(S)-HETE levels modestly, but non-significantly, increased (Figure 1D). 5(S)-HETE levels remained unchanged. To identify the 15-LOX isoform responsible for IL-4-induced 15(S)-HETE and 13(S)-HODE production, we used siRNAs to KD ALOX15 and ALOX15B. Macrophages were transfected with control, ALOX15, or ALOX15B siRNAs and stimulated with IL-4 for 48 h. Western analysis (Figures 1E,F) and mRNA expression (Figure S1 in Supplementary Material) of 15-LOXs confirmed KD efficiencies. 15(S)-HETE and 13(S)-HODE production significantly decreased in IL-4-treated ALOX15 KD but not ALOX15B KD macrophages, while 12(S)-HETE levels modestly but non-significantly decreased in IL-4-treated ALOX15 KD but not ALOX15B KD macrophages. These data indicate a predominant role of ALOX15 in 15(S)-HETE and 13(S)-HODE production following IL-4 stimulation (Figure 1G).

### KD of ALOX15 and ALOX15B Inhibits SREBP-2 Activation

**Figure 2:**
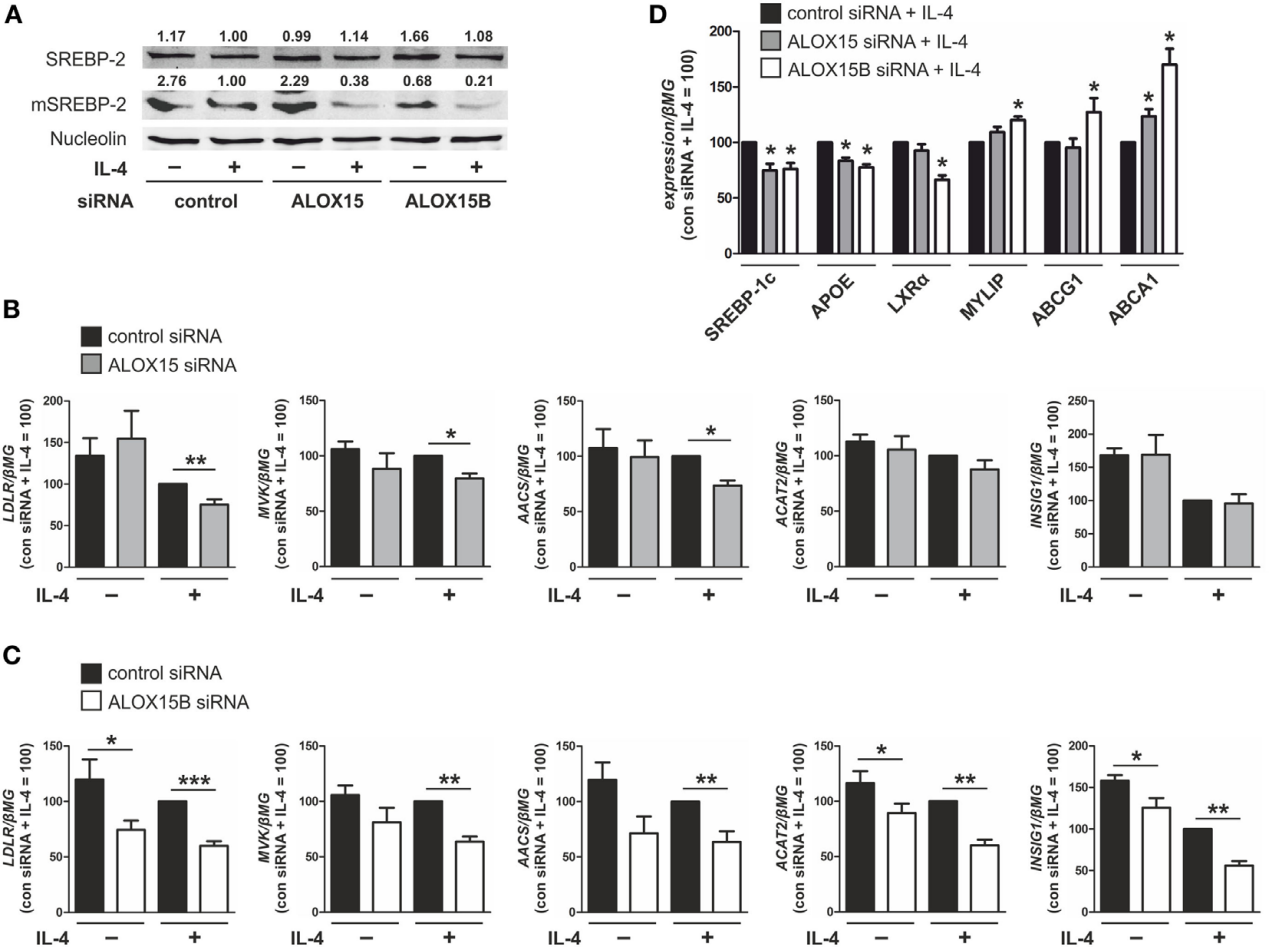
Knockdown of arachidonate 15-lipoxygenase (ALOX15) and arachidonate 15-lipoxygenase, type B (ALOX15B) inhibits sterol regulatory element binding protein (SREBP)-2 activation. **(A)** Representative Western analysis of SREBP-2 and mature N-terminal SREBP-2 (mSREBP-2) in macrophages transfected with control, ALOX15, or ALOX15B siRNAs 24 h prior to treatment with interleukin-4 (IL-4) for 48 h. **(B–D)** mRNA expression of **(B,C)** low density lipoprotein receptor (LDLR), mevalonate kinase (MVK), acetoacetyl-CoA synthetase (AACS), acetyl-CoA acetyltransferase 2 (ACAT2), insulin-induced gene 1 (INSIG1) **(D)** and SREBP-1c, apolipoprotein E (APOE), liver X receptor (LXR)α, myosin regulatory light chain interacting protein (MYLIP), ABCG1, ABCA1 in macrophages transfected with control and ALOX15 **(B,D)** or ALOX15B **(C,D)** siRNAs 24 h prior to treatment with IL-4 for 48 h. *P*-values were calculated using two-tailed Student’s *t*-test. *P < 0.05, **P < 0.01, ***P < 0.001. Data are mean values ± SE of at least three independent experiments.

Cellular cholesterol levels are reciprocally regulated by liver X receptors (LXRs) and the ER membrane-bound transcription factor sterol regulatory element (SRE) binding protein (SREBP)-2 (21). When ER membrane cholesterol levels fall below ~5% of total ER lipids, the sterol-sensing SREBP cleavage-activating protein (SCAP) escorts SREBP-2 to the Golgi for processing into the active mature N-terminal SREBP-2 (mSREBP-2) transcription factor to activate genes involved in cholesterol synthesis and uptake to restore cholesterol homeostasis (22). By contrast, LXR activity is normally induced under conditions of cellular cholesterol excess to promote efflux and restore cholesterol homeostasis (23). To investigate the role of 15-LOXs in macrophage cholesterol homeostasis we first used Western analysis to assess SREBP-2 activation in ALOX15- and ALOX15B-silenced macrophages following IL-4 stimulation. Untreated control siRNA-transfected macrophages contained mSREBP-2, which decreased following IL-4 treatment. Levels of mSREBP-2 in unstimulated ALOX15 KD macrophages were comparable to control macrophages but further decreased following IL-4 stimulation. Levels of mSREBP-2 in both unstimulated and IL-4-treated cells were strongly reduced in ALOX15B KD compared to control siRNA-transfected macrophages (Figure 2A). These data show that KD of ALOX15B reduces levels of mSREBP-2 in both untreated and IL-4 stimulated macrophages, whereas KD of ALOX15 only reduces mSREBP-2 levels following IL-4.

Next, we assessed gene expression of SREBP-2 target genes in ALOX15 and ALOX15B KD macrophages. We found several canonical SREBP-2 target genes to be reduced in KD macrophages. mRNA levels of low density lipoprotein receptor (LDLR), mevalonate kinase (MVK), and acetoacetyl-CoA synthetase (AACS) were reduced following IL-4 stimulation in ALOX15 and ALOX15B KD macrophages, while levels of acetyl-CoA acetyltransferase 2 and insulin-induced gene 1 (INSIG1) were reduced in ALOX15B but not ALOX15 KD macrophages compared to control siRNA-transfected macrophages (Figures 2B,C) (24, 25). LXR target gene expression following IL-4 stimulation was differentially modulated in KD macrophages as mRNA levels of both SREBP-1c and apolipoprotein E (APOE) were significantly decreased in KD macrophages while LXRα, which is subject to autoregulation in human macrophages (26), was reduced only in ALOX15B KD cells. Myosin regulatory light chain interacting protein (MYLIP), ATP-binding cassette transporter (ABC) G1, and ABCA1 gene expression were all significantly increased in ALOX15B KD macrophages. Only ABCA1 was increased in IL-4-stimulated ALOX15 KD cells (Figure 2D). Analysis of SREBP-2 processing as well as SREBP-2 and LXR target gene expression in KD macrophages suggests a preferential role of ALOX15B over ALOX15 in modulating cholesterol homeostasis.

### Cholesterol Levels in 15-LOX-Suppressed Macrophages

**Figure 3:**
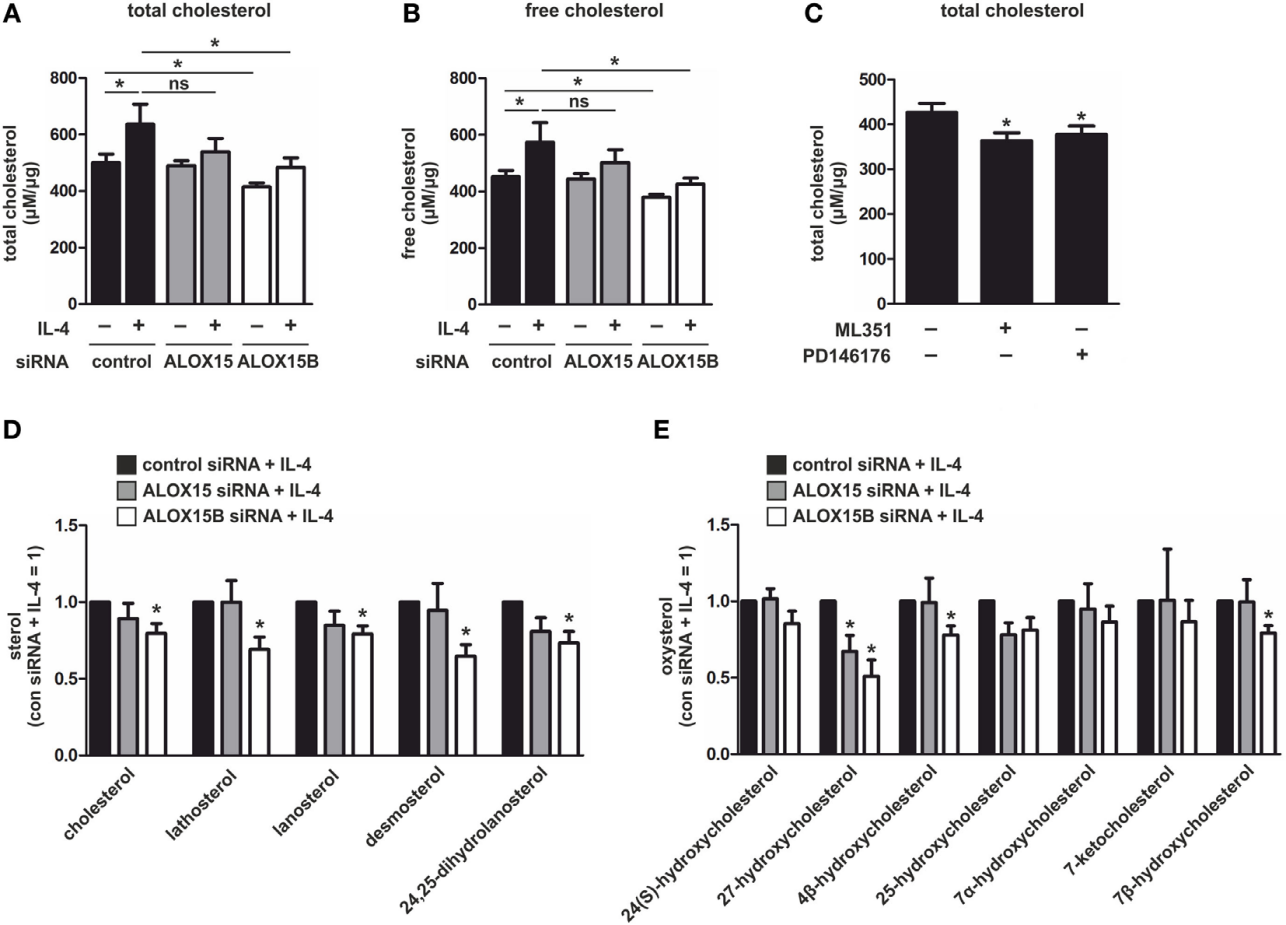
Sterol levels in interleukin-4 (IL-4)-stimulated macrophages. **(A,B)** Total **(A)** and free **(B)** cholesterol in macrophages transfected with control, arachidonate 15-lipoxygenase (ALOX15), or arachidonate 15-lipoxygenase, type B (ALOX15B) siRNAs 24 h prior to treatment with IL-4 for 48 h. *P*-values were calculated using one-way ANOVA with Bonferroni *post hoc* means comparisons with significance level set at 0.05. **(C)** Total cholesterol in macrophages treated with ML351 or PD146176 for 24 h. *P*-values were calculated using two-tailed Student’s *t*-test. **(D)** Cholesterol and non-cholesterol sterols **(E)** and oxysterols in macrophages transfected with control, ALOX15, or ALOX15B siRNAs 24 h prior to treatment with IL-4 for 48 h measured by gas chromatography. *P*-values were calculated using one-way ANOVA with Bonferroni *post hoc* means comparisons with significance level set at 0.05. *P < 0.05. Data are mean values ± SE of at least three independent experiments.

To determine whether the altered expression of cholesterol regulatory genes in 15-LOX KD macrophages translate to changes in cholesterol content, we measured total cellular cholesterol using a cholesterol fluorescence assay. Cholesterol content increased in control siRNA-transfected macrophages following 48 h stimulation with IL-4 (Figure 3A). Compared to control cells, both untreated and IL-4-stimulated ALOX15B KD macrophages showed significant reductions in cholesterol content. Total cellular cholesterol was modestly, but not significantly, reduced following IL-4 stimulation in ALOX15 KD cells. When overexpressed in cholesterol-loaded macrophages, ALOX15 oxygenated intracellular cholesteryl esters leading to increased CE hydrolase activity and reduced intracellular cholesteryl ester accumulation (8, 13, 14). Although KD of 15-LOXs reduced total cholesterol levels, we questioned whether cholesteryl ester levels remained elevated in KD macrophages, which might impact SREBP-2 signaling. Similar to total cholesterol, IL-4 stimulation increased free cholesterol in control siRNA-transfected macrophages but was significantly reduced in ALOX15B KD macrophages (Figure 3B). Free cholesterol levels were modestly, but not significantly reduced, in ALOX15 KD cells following IL-4 stimulation. Although both untreated and IL-4-stimulated ALOX15B KD cells had reduced levels of total and free cholesterol compared to control macrophages, esterified cholesterol levels remained low and were not significantly different compared to control and ALOX15 KD macrophages (data not shown). To confirm the preferential role for ALOX15B over ALOX15 in cellular cholesterol homeostasis we measured total cholesterol in naïve macrophages, which constitutively express ALOX15B protein but not ALOX15 (Figure 1B), following treatment with 15-LOX inhibitors. Treatment with ML351 and PD146176 reduced total cholesterol content 15 and 11%, respectively, after 24 h (Figure 3C). Collectively, silencing ALOX15B reduced cellular cholesterol content in untreated and IL-4-stimulated macrophages while treating with 15-LOX inhibitors reduced cholesterol content in macrophages expressing ALOX15B but not ALOX15. These observations suggest a novel role for ALOX15B in regulating cholesterol homeostasis.

In addition to cholesterol, SREBP-2 activation is inhibited by intermediates in the cholesterol-biosynthetic pathway and oxysterols (23). Binding of cholesterol precursors to SCAP triggers its binding to the ER transmembrane protein insulin-induced gene (INSIG), retaining it in the ER and preventing transport of SREBP-2 to the Golgi for processing and subsequent transcriptional activation. By contrast, oxysterols such as 25-hydroxycholesterol (HC) and 27-HC directly bind INSIG blocking transport of the SCAP/SREBP-2 complex to the Golgi (27). To determine whether increased levels of cholesterol intermediates or oxysterols reduced SREBP-2 activation and modulated LXR target gene expression in 15-LOX KD macrophages we performed a detailed lipidomic analysis by GC. Confirming the results of our cholesterol fluorescence assays, IL-4-stimulated ALOX15B KD macrophages contained significantly less cholesterol than IL-4-stimulated control cells. Cholesterol was modestly, but not significantly, reduced in IL-4-stimulated ALOX15 KD macrophages. IL-4-stimulated ALOX15B KD macrophages also contained significantly lower levels of cholesterol intermediates lathosterol, lanosterol, desmosterol, and 24,25-dihydrolanosterol compared to stimulated control cells (Figure 3D). The oxysterol ligand 27-HC, implicated in regulating LXR activity (23), was significantly reduced in both IL-4-stimulated ALOX15B and ALOX15 KD macrophages. 4β-hydroxycholesterol and 7β-hydroxycholesterol were significantly reduced in ALOX15B KD macrophages while 25-HC was non-significantly reduced in both IL-4-stimulated ALOX15B and ALOX15 KD macrophages compared to control cells (Figure 3E). Collectively, these results suggest SREBP-2 inhibition and enhanced expression of LXR target genes ABCA1, ABCG1, and MYLIP in IL-4-stimulated ALOX15B KD macrophages are unlikely mediated through increased concentrations of SREBP-2- or LXR-modulating cholesterol-biosynthetic pathway intermediates or oxysterols measured in our lipidomic analysis.

### Inhibiting 15-Lipoxygenase Activity Reduces SREBP-2 Binding to SREs

**Figure 4:**
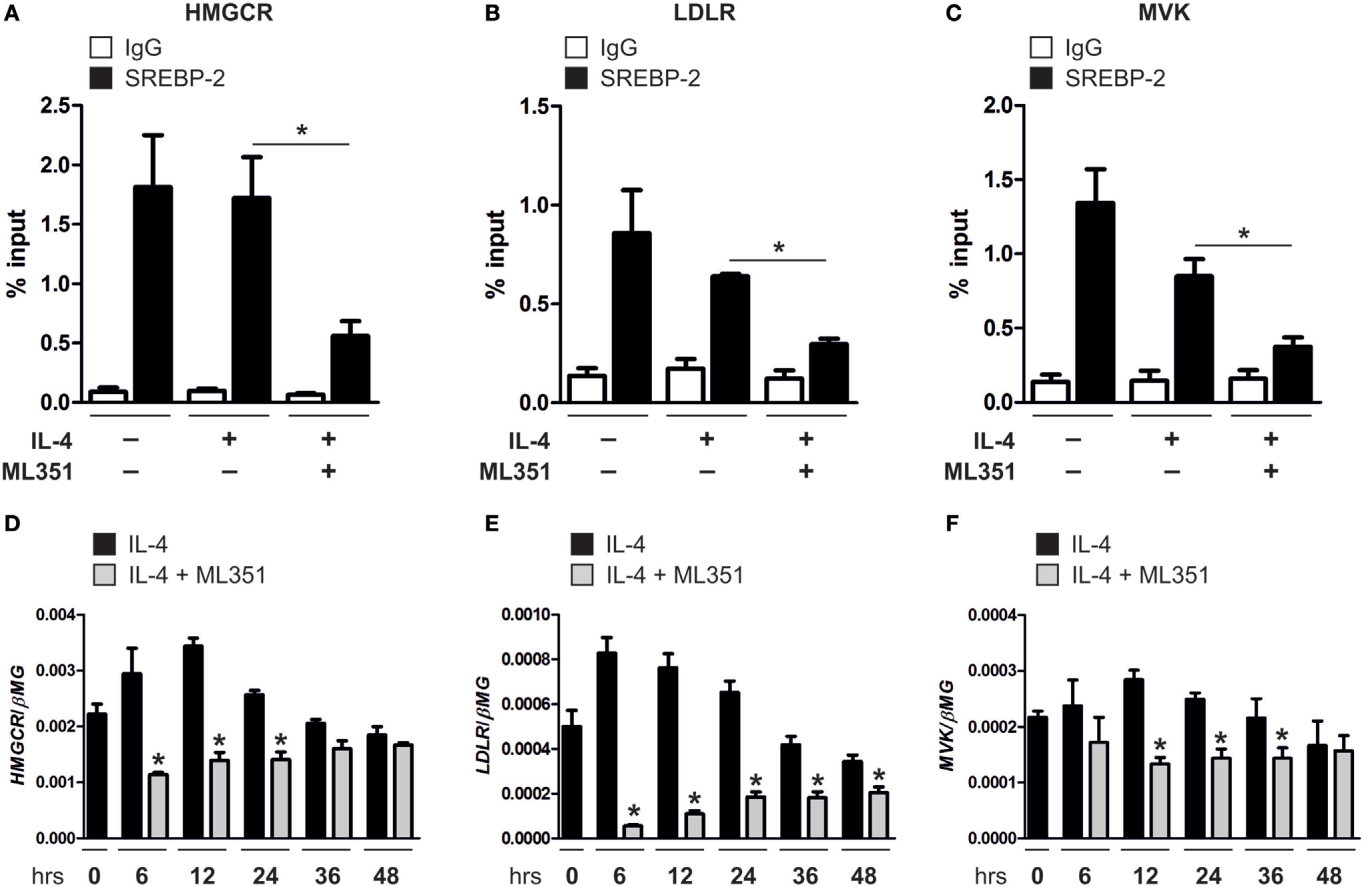
Inhibiting 15-lipoxygenase activity reduces sterol regulatory element binding protein (SREBP)-2 binding to sterol regulatory elements. **(A–C)** Chromatin immunoprecipitation (ChIP) analysis of SREBP-2 binding to promoter of **(A)** 3-hydroxy-3-methylglutaryl-CoA reductase (HMGCR), **(B)** low density lipoprotein receptor (LDLR), and **(C)** mevalonate kinase (MVK) in untreated or macrophages stimulated with interleukin-4 (IL-4) and ML351 or IL-4 alone for 16 h. **(D–F)** mRNA expression of **(D)** HMGCR, **(E)** LDLR, and **(F)** MVK in macrophages stimulated with IL-4 and ML351 or IL-4 alone in a time-dependent manner. *P*-values were calculated using one-way ANOVA with Bonferroni *post hoc* means comparisons with significance level set at 0.05. *P < 0.05. Data are mean values ± SE of four independent experiments.

Cholesterol synthesis and uptake pathways are regulated at the transcriptional level, whereby in cholesterol-depleted conditions mature SREBP-2 enters the nucleus and binds SREs in the promoter of target genes to induce gene expression and restore cholesterol homeostasis. To determine whether inhibiting 15-LOXs directly inhibits SREBP-2 binding to SREs, we performed ChIP assays in human macrophages. SREBP-2 binding was enriched in SREs within the promoters of 3-hydroxy-3-methylglutaryl-CoA reductase (HMGCR), LDLR, and MVK of untreated macrophages and following 16 h stimulation with IL-4, but reduced in cells co-incubated with the 15-LOX inhibitor ML351 (Figures 4A–C). This reduced SREBP-2 binding to SREs in macrophages coincided with lower expression of HMGCR, LDLR, and MVK in macrophages co-cultured with IL-4 and ML351 compared with IL-4 alone (Figures 4D–F).

Since our knowledge of the biological roles of the lipid products derived from 15-LOX enzymatic activity remain limited, we assessed whether 15-LOX-derived lipid metabolites modulate SREBP-2 activation by performing rescue experiments with 15-LOX inhibitors ML351 and PD146176. Macrophages were pre-treated with or without ML351 or PD146176, then stimulated with IL-4 alone or co-incubated with exogenous arachidonic acid-, linoleic acid-, or docosahexaenoic acid-derived 15-LOX metabolites 15(S)-HETE, 13(S)-HODE, or 17(S)-HDHA, respectively. Reduced SREBP-2 target gene expression in ML351 or PD146176 and IL-4 co-stimulated macrophages was not rescued by supplementing with exogenous 15(S)-HETE, 13(S)-HODE, or 17(S)-HDHA (data not shown) suggesting the loss of 15-LOX-mediated non-esterified lipid products in KD macrophages are likely not responsible for reduced SREBP-2 activation.

### ALOX15 and ALOX15B Modulate CCL17 Expression in an SREBP-2-Dependent Manner

**Figure 5:**
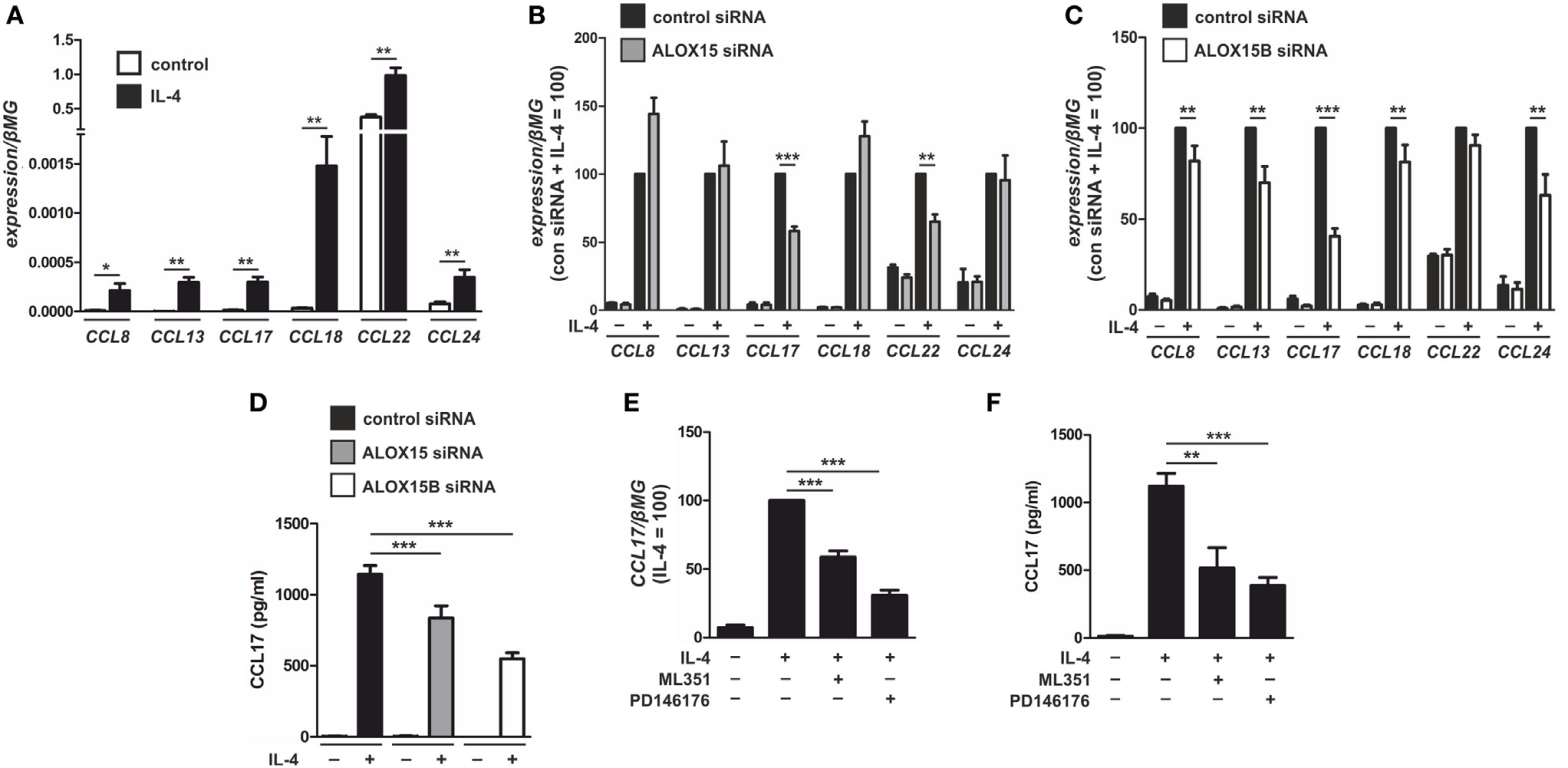
Chemokine modulation by 15-LOX isoforms in interleukin-4 (IL-4)-stimulated macrophages. **(A)** mRNA expression of CCL8, CCL13, CCL17, CCL18, CCL22, and CCL24 in primary human macrophages treated with 20 ng/ml IL-4 for 48 h. **(B,C)** Chemokine mRNA expression in macrophages transfected with control and arachidonate 15-lipoxygenase (ALOX15) **(B)** or arachidonate 15-lipoxygenase, type B (ALOX15B) **(C)** siRNAs 24 h prior to treatment with IL-4 for 48 h. **(D)** CCL17 protein concentrations in culture medium of macrophages transfected with control, ALOX15, or ALOX15B siRNAs 24 h prior to treatment with IL-4 for 48 h. **(E,F)** CCL17 mRNA expression **(E)** and protein concentrations **(F)** from macrophages treated with either ML351 (10 µM) or PD146176 (10 µM) for the final 24 h post IL-4 stimulation. *P*-values were calculated using one-way ANOVA with Bonferroni *post hoc* means comparisons with significance level set at 0.05. *P < 0.05, **P < 0.01, ***P < 0.001. Data are mean values ± SE of at least three independent experiments.

Next, we assessed the functional consequence of altered SREBP-2 signaling in AAMs. Gene ontology data derived from global transcriptome analysis identified chemokines as one of the most overrepresented functional categories in IL-4-stimulated human monocyte-derived macrophages (3). Since previous reports showed ALOX15 (28) and ALOX15B (29) modulate chemokine production we next sought to identify SREBP-2 transcriptional target genes among the IL-4-induced chemokines in AAMs. Corroborating previous studies (1, 3, 30), IL-4 stimulation of human macrophages increased mRNA levels of C-C motif chemokine ligand (CCL) 8, CCL13, CCL17, CCL18, CCL22, and CCL24 (Figure 5A). To determine the impact of each 15-LOX isoform on IL-4-induced chemokine production we again used siRNAs to KD ALOX15 and ALOX15B. The expression of typical IL-4 target genes in macrophages, such as mannose receptor C-type 1, transglutaminase 2, or peroxisome proliferator activated receptor gamma remained unaffected by IL-4 stimulation in ALOX15 and ALOX15B KD macrophages compared to control siRNA-transfected macrophages (Figure S2A in Supplementary Material), suggesting that 15-LOX-silencing did not affect global IL-4-induced gene expression. With respect to IL-4-induced chemokines, CCL17 and CCL22 mRNA expression levels were reduced in ALOX15-silenced macrophages (Figure 5B), while CCL8, CCL13, CCL17, CCL18, and CCL24 levels were reduced in ALOX15B KD cells (Figure 5C) compared to IL-4-stimulated control siRNA-transfected macrophages. In IL-4-stimulated ALOX15/ALOX15B double KD macrophages only CCL17 mRNA levels were reduced compared to IL-4-stimulated control macrophages (Figure S2B in Supplementary Material). CCL17 levels were undetectable in supernatants of unstimulated macrophages, increased following IL-4 stimulation, but remained lower in ALOX15 and ALOX15B KD macrophages compared to control siRNA-transfected macrophages (Figure 5D). To confirm regulation of CCL17 by ALOX15 and ALOX15B, macrophages were treated with PD146176 or ML351 for the final 24 h post IL-4 stimulation. Both ML351 and PD146176 decreased CCL17 mRNA (Figure 5E) and protein levels (Figure 5F) in response to IL-4 stimulation. Conclusively, attenuating 15-LOX expression did not generally affect IL-4 gene expression but rather uniquely impacted IL-4-induced CCL17 expression.

**Figure 6:**
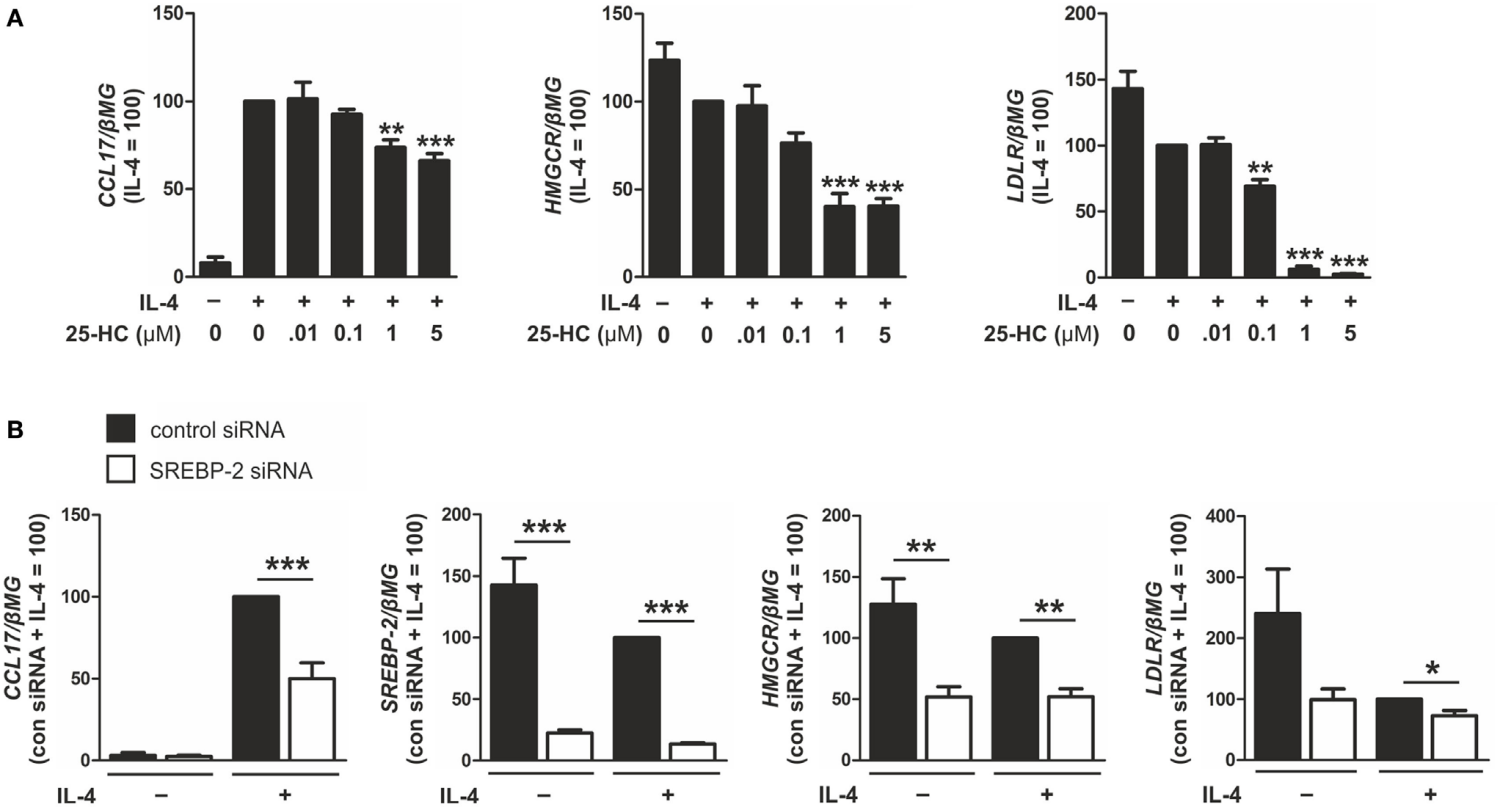
Inhibiting sterol regulatory element binding protein (SREBP)-2 activation reduces CCL17 expression. **(A)** mRNA expression of CCL17, 3-hydroxy-3-methylglutaryl-CoA reductase (HMGCR), and low density lipoprotein receptor (LDLR) in macrophages treated with indicated concentrations of 25-HC and interleukin-4 (IL-4) for 24 h. **(B)** mRNA expression of CCL17, SREBP2, HMGCR, and LDLR in macrophages transfected with control or SREBP2 siRNA 24 h prior to treatment with IL-4 for 48 h. *P*-values were calculated using one-way ANOVA with Bonferroni *post hoc* means comparisons with significance level set at 0.05. *P < 0.05, **P < 0.01, ***P < 0.001. Data are mean values ± SE of at least three independent experiments.

To determine whether CCL17 gene expression is modulated by SREBP-2 we next assessed expression after either inhibiting or activating SREBP-2. To inhibit SREBP-2 activation, macrophages were treated with 25-HC or transfected with siRNAs targeting SREBP2. Treatment with 25-HC, which binds INSIG and blocks transport of the SCAP/SREBP-2 complex to the Golgi (27), dose-dependently reduced mRNA expression of CCL17 and the SREBP-2 target genes LDLR and HMGCR in IL-4-stimulated macrophages (Figure 6A). Next, macrophages were transfected with control or SREBP2-targeting siRNAs for 48 h, then stimulated with IL-4. Silencing SREBP2 reduced SREBP2 as well as CCL17, HMGCR, and LDLR mRNA levels in IL-4-stimulated macrophages compared to control cells (Figure 6B).

**Figure 7:**
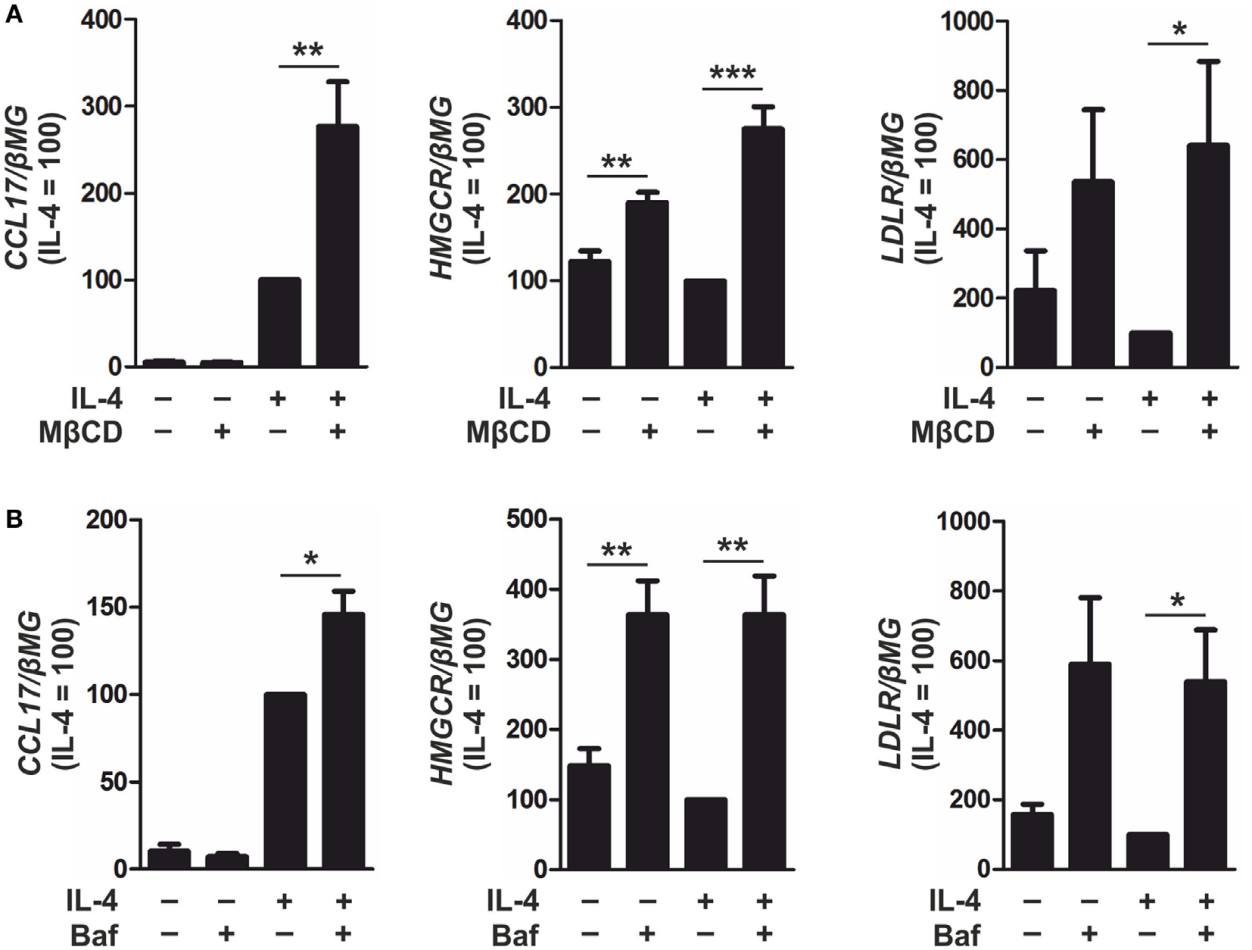
Inducing sterol regulatory element binding protein (SREBP)-2 activation enhances CCL17 expression. **(A)** mRNA expression of CCL17, 3-hydroxy-3-methylglutaryl-CoA reductase (HMGCR), and low density lipoprotein receptor (LDLR) in macrophages cultured overnight in serum-free RPMI containing MβCD (1 mM) then treated with interleukin-4 (IL-4) in serum-free media for 6 h. **(B)** mRNA expression of CCL17, HMGCR, and LDLR in macrophages treated with IL-4 and Bafilomycin A1 (100 nM) for 24 h. *P*-values were calculated using one-way ANOVA with Bonferroni *post hoc* means comparisons with significance level set at 0.05. *P < 0.05, **P < 0.01, ***P < 0.001. Data are mean values ± SE of at least three independent experiments.

To enhance SREBP-2 activation, macrophages were incubated with methyl-β-cyclodextrin (MβCD) to deplete cellular cholesterol (22) or Bafilomycin A1 to inhibit lysosomal acidification and sequester cholesterol in the endosomal/lysosomal compartment (31). Overnight culture with MβCD followed by IL-4 stimulation increased CCL17, HMGCR, and LDLR mRNA levels compared with IL-4-stimulated control macrophages (Figure 7A). In addition, co-treatment of IL-4 and Bafilomycin A1 increased expression of CCL17, HMGCR, and LDLR compared with macrophages stimulated with IL-4 alone (Figure 7B). These results suggest that IL-4-induced CCL17 gene expression is modulated in an SREBP-2-dependent manner in primary human macrophages.

To examine whether SREBP-2 directly activates CCL17 expression in response to IL-4 stimulation we performed bioinformatics analysis for putative SREs in the CCL17 promoter and enhancer regions and performed ChIP assays. We failed to detect SREBP-2 enrichment within the CCL17 promoter or within enhancer regions following IL-4 stimulation (data not shown). The locations of enhancers are highly variable with respect to their target genes. Enhancers do not necessarily act on the respective closest promoter and can bypass neighboring genes to regulate genes located more distantly along a chromosome. Therefore, it cannot conclusively be ruled out that SREBP-2 binds an unidentified enhancer region to potentiate CCL17 expression (32).

### ALOX15 and ALOX15B-Mediated Changes in Chemokine Production Alter T Cell Migration

**Figure 8:**
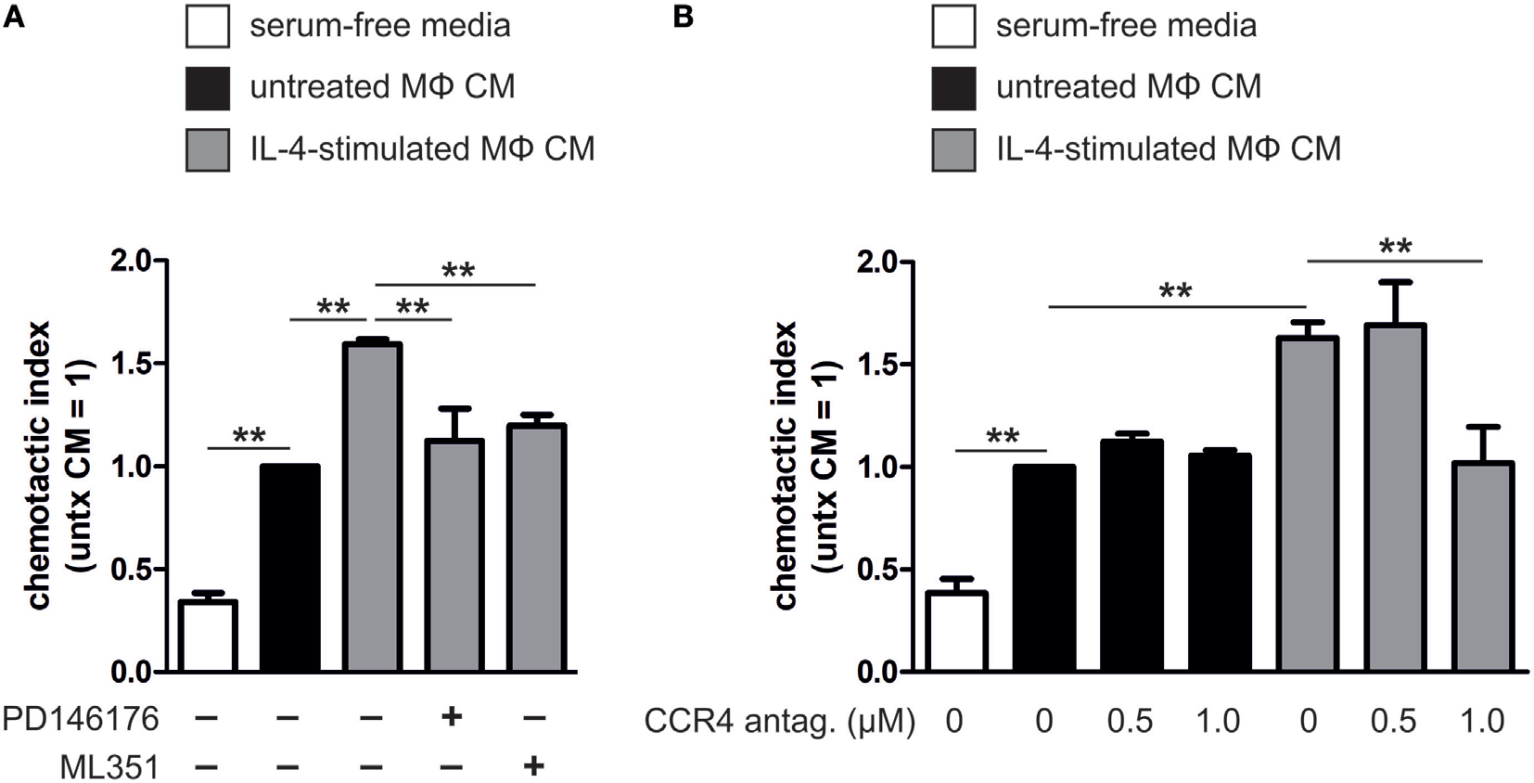
Migration of HUT78 cells to macrophage conditioned media (CM). **(A)** Migration of HUT78 cells to serum-free media or CM from untreated or interleukin-4 (IL-4)-stimulated macrophages co-incubated with or without PD146176 and ML351. **(B)** Migration of HUT78 cells pre-treated with or without CC chemokine receptor 4 (CCR4) antagonist to CM from untreated or IL-4-stimulated human primary macrophages. *P*-values were calculated using one-way ANOVA with Bonferroni *post hoc* means comparisons with significance level set at 0.05. **P < 0.01. Data are mean values ± SE of at least three independent experiments.

Next, we addressed the functional significance of ALOX15- and ALOX15B-mediated chemokine regulation in macrophages by performing chemotactic migration assays of HUT78 cells expressing the high-affinity CCL17 receptor CCR4 (33, 34). As a first experiment, we measured migration of HUT78 cells to macrophage CM. Compared to serum-free media, CM from unstimulated macrophages enhanced HUT78 cell migration (Figure 8A). Migration to CM from IL-4-stimulated macrophages was further enhanced compared to unstimulated macrophage CM, but was completely abated by the addition of ML351 or PD146176. To assess the chemotactic impact of endogenously produced CCL17 in macrophage CM we pre-treated HUT78 cells with a CCR4 antagonist, then assessed migration to CM from IL-4-stimulated or control macrophages. HUT78 cell migration to unstimulated macrophage CM was unaffected by pre-treatment with the CCR4 antagonist but was diminished to IL-4-stimulated macrophage CM when pre-treated with 1 µM CCR4 antagonist (Figure 8B). Taken together, these data indicate CCL17 released from IL-4-stimulated macrophages in a 15-LOX-dependent manner mediates HUT78 cell migration *via* CCR4.

### ALOX15B Expression Correlates With Clinical Parameters in Asthmatic Patients

**Figure 9:**
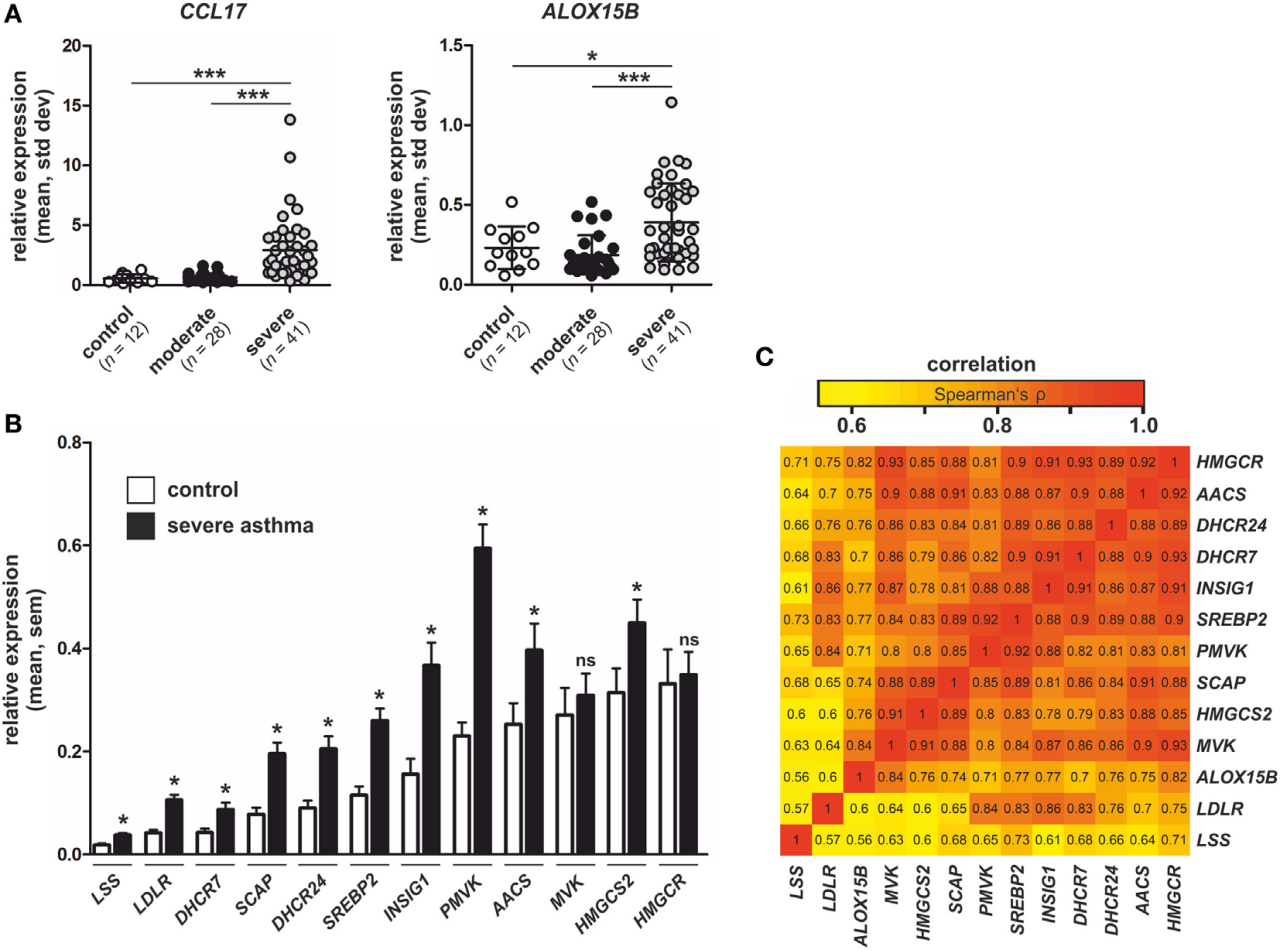
Expression of CCL17, arachidonate 15-lipoxygenase, type B (ALOX15B), and sterol regulatory element binding protein (SREBP)-2 target genes in asthmatic subjects. **(A)** CCL17 and ALOX15B expression in bronchial alveolar lavage (BAL) cells isolated from healthy controls (*n* = 12), moderate asthmatics (*n* = 28), and severe asthmatics (*n* = 41). *P*-values were calculated using one-way ANOVA with Bonferroni *post hoc* means comparisons with significance level set at 0.05. *P < 0.05, ***P < 0.001. **(B)** Expression of SREBP-2 target gene lanosterol synthase (LSS), low density lipoprotein receptor (LDLR), 7-dehydrocholesterol reductase (DHCR7), SREBP cleavage-activating protein (SCAP), 24-dehydrocholesterol reductase (DHCR24), SREBP2, insulin-induced gene 1 (INSIG1), phosphomevalonate kinase (PMVK), acetoacetyl-CoA synthetase (AACS), mevalonate kinase (MVK), 3-hydroxy-3-methylglutaryl-CoA synthase 2 (HMGCS2), and 3-hydroxy-3-methylglutaryl-CoA reductase (HMGCR) in BAL cells isolated from control subjects and severe asthmatics. *P*-values were calculated using two-tailed Student’s *t*-test. *P < 0.05. **(C)** Pairwise correlations of gene expression in BAL cells isolated from severe asthmatics. The heat map indicates pairwise correlation strength (Spearman’s ρ) between ALOX15B and SREBP-2 target genes.

Since our data links macrophage ALOX15B to CCL17 production and T cell migration, we reasoned whether these findings might be relevant to human asthma. In asthma, increased CCL17 production by lung macrophages attract CCR4+ Th2 T lymphocytes, which play a central role in orchestrating airway inflammation (35–37). Since CCL17 levels are elevated in sputum and BAL of asthmatic patients (35, 38), we explored ALOX15B expression in datasets of asthmatic patients in the NCBI Gene Expression Omnibus (GEO) repository. We identified a gene expression dataset of human BAL cells from control and asthmatic subjects (GEO accession number GSE74986). Analysis of the dataset revealed increased CCL17 and ALOX15B expression in BAL cells isolated from severe asthmatics compared to moderate asthmatic patients and healthy controls (Figure 9A). ALOX15 expression in BAL cells was not significantly different between control and asthmatic subjects (data not shown). Since our *in vitro* data showed silencing or inhibiting the 15-LOX isoforms in human macrophages impaired SREBP-2 signaling and reduced target gene expression, we assessed the expression of SREBP-2 target genes in severe asthmatics in which ALOX15B expression was significantly increased. We found expression of several canonical SREBP-2 target genes, including lanosterol synthase, LDLR, 7-dehydrocholesterol reductase, SCAP, 24-dehydrocholesterol reductase, SREBP-2, INSIG1, phosphomevalonate kinase (PMVK), AACS, and 3-hydroxy-3-methylglutaryl-CoA synthase 2 (HMGCS2) were increased in BAL cells isolated from severe asthmatics compared to healthy controls (Figure 9B). To assess the pairwise correlation strength between ALOX15B and SREBP-2 target gene expression in BAL cells isolated from severe asthmatics, pairwise correlations were determined using Spearman’s rank coefficient. As expected, strong positive correlations existed between all SREBP-2 target genes examined (Figure 9C). Consistent with our *in vitro* findings which showed impaired SREBP-2 processing, reduced SREBP-2 binding to SREs and subsequent target gene expression in ALOX15B-suppressed macrophages, the expression of SREBP-2 target genes exhibited strong positive correlations with ALOX15B in BAL cells isolated from severe asthmatics. Collectively, these data support the notion that enhanced ALOX15B expression in macrophages contribute to increased CCL17 production in an SREBP-2-dependent manner, and suggest that alternative therapies to reduce CCL17 levels and subsequent T cell migration by targeting the 15-LOX enzymes may offer therapeutic potential for treating chronic airway diseases such as asthma.

## Discussion

Although not completely understood, the biological roles of the 15-LOX isoforms ALOX15 and ALOX15B contribute to various macrophage functions, including resolution of inflammation through synthesis of bioactive lipid mediators (6, 39), efferocytosis and tissue remodeling (11, 40, 41), as well as chemokine production (29, 42). Cellular cholesterol and intermediates in the cholesterol-biosynthetic pathway, which are collectively regulated by the transcription factor SREBP-2, are instrumental in orchestrating diverse cellular processes including an ever-expanding role of macrophage innate immune responses (23, 43). Since previous publications have reported cholesterol-modifying and mobilizing activities of 15-LOX in macrophages (8, 12–14, 44), we aimed to better understand the role of 15-LOXs in cellular cholesterol homeostasis and AAM function. Through a combination of inhibitors and siRNAs to suppress the 15-LOX isoforms, our findings suggest ALOX15B, and to a lesser extent ALOX15, regulate cholesterol homeostasis and CCL17 production in human macrophages in an SREBP-2-dependent manner. Silencing or inhibiting the 15-LOX isoforms impaired SREBP-2 signaling by inhibiting SREBP-2 processing, reduced SREBP-2 binding to SREs and subsequent target gene expression, and decreased cellular cholesterol, cholesterol intermediates, and oxysterols.

How 15-LOXs modulate the SREBP-2 regulatory system is unknown. The ER plays a central role in the feedback regulation of cholesterol homeostasis. When ER membrane cholesterol falls below ~5% of total ER lipids, the SCAP/SREBP-2 complex is escorted to the Golgi for processing into an active transcription factor to activate genes involved in cholesterol synthesis and uptake (22). As a consequence, cholesterol levels rise allowing sterols to bind SCAP and preventing the SCAP/SREBP-2 complex from leaving the ER (22). As a result, SREBP-2 is no longer processed and cellular cholesterol homeostasis is restored. Recent studies provide clues about cellular cholesterol monitoring and transport. The majority (60–90%) of a cell’s total cholesterol is distributed within distinct cholesterol pools in the PM at a cholesterol:phospholipid mole ratio of 0.50–0.25 (15, 17). The cholesterol within one of these pools is continuously transported from PM to ER, where cholesterol is kept at a much lower cholesterol:phospholipid mole ratio (~0.05) (22, 45). This continuous transport allows the ER to constantly monitor PM cholesterol levels and rapidly respond to small declines by activating SREBP-2 to maintain cholesterol homeostasis (45). The distribution of cholesterol within cellular membranes is not fixed but is dictated by the relative abundance and affinity of various phospholipids and sphingolipids, which can alter the cholesterol:phospholipid mole ratios by as much as 10-fold (16). Macrophages derived from 12/15-lipoxygenase deficient mice showed altered cell membrane structure, increased levels of PE, and reduced levels of PC (10). As the most abundant phospholipid in eukaryotic cells, PC positively influences the incorporation of cholesterol in membranes (46). As a precursor for the synthesis of sphingomyelin, PC also indirectly affects sphingomyelin-enriched nanodomains shown to alter intracellular cholesterol distribution (47). Whether 15-LOXs regulate membrane cholesterol levels through altering cellular phospholipid and sphingolipid levels is not known. Co-incubating KD macrophages with 15-LOX-derived lipid products 15(S)-HETE, 13(S)-HODE, and 17(S)-HDHA failed to restore SREBP-2 target gene expression. This is not surprising, considering the fate of exogenous 15-LOX products differ from those generated endogenously. Endogenous 15(S)-HETE is present primarily as PE-esterified HETE generated by direct oxidation of PE and differs from exogenous 15(S)-HETE, which is not esterified into any phospholipid pool suggesting the requirement for 15-LOXs cannot be replicated by exogenous 15-LOX-derived products (48). Collectively, the extent to which ALOX15B plays a role in membrane remodeling thereby altering cholesterol:phospholipid mole ratios, and whether such disturbances alter SREBP-2 activation and cellular cholesterol homeostasis is unknown and will be the subject of future studies.

In addition to SREBP-2, cellular cholesterol homeostasis is maintained by LXR transcription factors which function to eliminate excess cholesterol (23). Although SREBP-2 and LXR control antagonistic aspects of cellular sterol homeostasis, they are intimately connected as demonstrated recently by Rong et al. in which deleting SREBP-2 reduced sterol synthesis and eliminated production of endogenous sterol ligands required for LXR activity and expression of the LXR target gene SREBP-1c (49). This corresponds well with our data in which IL-4-stimulated ALOX15B KD macrophages, which showed impaired SREBP-2 signaling, contained lower levels of endogenous LXR-activating ligands including cholesterol intermediates and oxysterols, and reduced expression of LXR target genes SREBP-1c, APOE, and LXRα. Interestingly, while SREBP-1c, APOE, and LXRα gene expression was reduced in IL-4-stimulated ALOX15B KD macrophages, expression of LXR target genes ABCA1 and ABCG1 were enhanced. Since LXR-independent regulatory mechanisms have been described for both ABCA1 and ABCG1, we cannot rule out their enhanced expression in ALOX15B KD macrophages may occur through mechanisms independent of LXR (50, 51).

The role of SREBPs as regulators of cholesterol and lipid homeostasis is firmly established. Recently, however, several studies have uncovered roles for SREBPs in key responses of the innate immune system in macrophages. Using a mouse with whole-body deficiency of Srebp-1a, researchers showed that Srebp-1a in macrophages activate the gene encoding the core inflammasome component Nlrp1a (43). Meanwhile Srebp-2 activation was shown to be responsible for LPS-induced Il1β, Il1α, and Il18 expression in murine macrophages (52) further supporting a link between cholesterol metabolism and innate immune responses. In murine alveolar type 2 cells, deletion of both Insig1 and Insig2 genes activated SREBPs and increased expression of Ccl17, further supporting a regulatory mechanism between SREBP and CCL17 (53). Of the many chemokines expressed in AAMs, the unique regulation of CCL17 by SREBP-2 is an interesting question warranting further investigation. The promoter of CCL17 contains several transcriptional regulation sites including signal transducer and activator of transcription (STAT) 6 binding sites and a nuclear factor-kappa B (NF-κB). Suggestively, CCL17 expression can be induced by Th2 cytokines *via* STAT6 but also through NF-κB activation in response to toll-like receptor activation (54–57). We were unable to detect SREBP-2 binding in the promoter of CCL17 in ChIP assays using human macrophages, which is in line with existing ChIP-seq data accessible through the Cistrome dataset browser^2^ in which SREBP-2 binding is also not observed in CCL17 promoters of numerous human cell lines. With this in mind, SREBP-2 may bind an unidentified enhancer region to potentiate induction of CCL17 by STAT6 or NF-κB. Moreover, due to its constitutive expression, SREBP-2 is uniquely positioned to regulate CCL17 as well as other potential target genes that may be differentially regulated within the spectrum of macrophage polarization states including classically and alternatively activated ones.

In summary, we identified a novel role for ALOX15B, and to a lesser extent ALOX15, in regulating cholesterol homeostasis and CCL17 production in human macrophages in an SREBP-2-dependent manner. SREBPs are implicated in numerous pathogenic processes such as ER stress, inflammation, and apoptosis, and in this way they contribute to chronic diseases including asthma, diabetes, and cancers (58). Uncovering links between 15-LOXs and SREBP-2 regulation provides new opportunities to regulate cholesterol and immune responses.

## Author Contributions

RS and BB managed the project. RS designed the experiments. RS, DL, EZ, and CA performed the experiments. RS, DN, EZ, SG, and BB analyzed the data. RS wrote the manuscript. RS, BB, and DN edited the manuscript.

## Conflict of Interest Statement

The authors declare that the research was conducted in the absence of any commercial or financial relationships that could be construed as a potential conflict of interest.

## Footnotes

^1^www.R-project.org (Accessed: April 19, 2018).

^2^http://cistrome.org (Accessed: January 16, 2018).

## Supplementary Material

Figure S1 Arachidonate 15-lipoxygenase (ALOX15) and arachidonate 15-lipoxygenase, type B (ALOX15B) mRNA expression in knockdown macrophages. mRNA expression of ALOX15 and ALOX15B in macrophages transfected with control and ALOX15 or ALOX15B siRNAs 24 h prior to treatment with interleukin-4 (IL-4) for 48 h. *P*-values were calculated using one-way ANOVA with Bonferroni *post hoc* means comparisons with significance level set at 0.05. ****P* < 0.001. Data are mean values ± SE of five independent experiments.

Figure S2 Gene expression in arachidonate 15-lipoxygenase (ALOX15) and arachidonate 15-lipoxygenase, type B (ALOX15B) knockdown macrophages. **(A)** mRNA expression of mannose receptor C-type 1 (MRC1), transglutaminase 2 (TGM2), and peroxisome proliferator activated receptor gamma (PPARG) in macrophages transfected with control, ALOX15, or ALOX15B siRNAs 24 h prior to treatment with interleukin-4 (IL-4) for 48 h. **(B)** Chemokine mRNA expression in macrophages transfected with control or ALOX15 and ALOX15B siRNAs 24 h prior to treatment with IL-4 for 48 h. *P*-values were calculated using one-way ANOVA with Bonferroni *post hoc* means comparisons with significance level set at 0.05. ****P* < 0.001. Data are mean values ± SE of at least three independent experiments.

## Abbreviations

AACS, acetoacetyl-CoA synthetase; AAM, alternatively activated macrophage; ACAT2, acetyl-CoA acetyltransferase 2; ALOX15, arachidonate 15-lipoxygenase; ALOX15B, arachidonate 15-lipoxygenase, type B; CE, cholesterol ester; DHCR7, 7-dehydrocholesterol reductase; DHCR24, 24-dehydrocholesterol reductase; HETE, hydroxyeicosatetraenoic acid; HMGCS2, 3-hydroxy-3-methylglutaryl-CoA synthase 2; HMGCR, 3-hydroxy-3-methylglutaryl-CoA reductase; HODE, hydroxyoctadecadienoic acid; HpETE, hydroperoxyeicosatetraenoic acid; 25-HC, 25-hydroxycholesterol; INSIG, insulin-induced gene; KD, knockdown; LDLR, low density lipoprotein receptor; LOX, lipoxygenase; LSS, lanosterol synthase; mSREBP-2, mature N-terminal SREBP-2; MVK, mevalonate kinase; PC, phosphatidycholine; PE, phosphatidylethanolamine; PI, phosphatidylinositol; PMVK, phosphomevalonate kinase; PUFA, polyunsaturated fatty acid; SCAP, SREBP cleavage-activating protein; SRE, sterol regulatory element; SREBP, sterol regulatory element binding protein.
